# Ochratoxin A Inhibits Mouse Embryonic Development by Activating a Mitochondrion-Dependent Apoptotic Signaling Pathway

**DOI:** 10.3390/ijms14010935

**Published:** 2013-01-07

**Authors:** Yan-Der Hsuuw, Wen-Hsiung Chan, Jau-Song Yu

**Affiliations:** 1 Department of Life Science, National Pingtung University of Science and Technology, Pingtung 912, Taiwan; E-Mail: hsuuw@yahoo.com.tw; 2 Department of Bioscience Technology and Center for Nanotechnology, Chung Yuan Christian University, Chung Li 320, Taiwan; 3 Department of Cell and Molecular Biology, Institute of Biomedical Sciences, Medical College of Chang Gung University, Tao-Yuan 333, Taiwan; E-Mail: yusong@mail.cgu.edu.tw

**Keywords:** ochratoxin A, blastocyst, apoptosis, development, ROS

## Abstract

Ochratoxin A (OTA), a mycotoxin found in many foods worldwide, causes nephrotoxicity, hepatotoxicity, and immunotoxicity, both *in vitro* and *in vivo*. In the present study, we explored the cytotoxic effects exerted by OTA on the blastocyst stage of mouse embryos, on subsequent embryonic attachment, on outgrowth *in vitro*, and following *in vivo* implantation via embryo transfer. Mouse blastocysts were incubated with or without OTA (1, 5, or 10 μM) for 24 h. Cell proliferation and growth were investigated using dual differential staining; apoptosis was measured using the terminal deoxynucleotidyl transferase-mediated dUTP nick-end labeling (TUNEL) assay; and embryo implantation and post-implantation development were assessed by examination of *in vitro* growth and the outcome of *in vivo* embryo transfer, respectively. Blastocysts treated with 10 μM OTA displayed a significantly increased level of apoptosis and a reduction in total cell number. Interestingly, we observed no marked difference in implantation success rate between OTA-pretreated and control blastocysts either during *in vitro* embryonic development (following implantation in a fibronectin-coated culture dish) or after *in vivo* embryo transfer. However, *in vitro* treatment with 10 μM OTA was associated with increased resorption of post-implantation embryos by the mouse uterus, and decreased fetal weight upon embryo transfer. Our results collectively indicate that *in vitro* exposure to OTA triggers apoptosis and retards early post-implantation development after transfer of embryos to host mice. In addition, OTA induces apoptosis-mediated injury of mouse blastocysts, via reactive oxygen species (ROS) generation, and promotes mitochondrion-dependent apoptotic signaling processes that impair subsequent embryonic development.

## 1. Introduction

Ochratoxin A (OTA) is a mycotoxin produced principally by ubiquitous strains of *Aspergillus* and *Penicillium* [1]. OTA is one of the most common food-contaminating mycotoxins, and is often isolated from beans, grains, cereals and spices. In addition, OTA has contaminated coffee, grape juice, wine, beer and bread [2]. It is very difficult to completely avoid dietary exposure to OTA because the chemical occurs widely in various food stuffs [3,4]. Therefore, it is important to study the adverse effects of OTA on humans. Previous studies have found that OTA is nephrotoxic and hepatotoxic, and causes neurodegenerative disease [5]. In addition, recent work has shown that OTA creates oxidative stress in several regions of the mouse midbrain and hippocampus, compromising brain development [6]. OTA is a potent carcinogen and induces tumors in the kidney, mammary gland, and liver [7–9]. A recent study found that OTA triggered apoptosis *via* activation of a mitochondrion-dependent pathway. OTA induces apoptosis by elevating ROS generation, by causing mitochondrial transmembrane potential to be lost *via* opening of mitochondrial pores, by triggering the release of cytochrome c, and by activating caspase [10]. Thus, although OTA appears to exert multiple biological actions, and is cytotoxic [4,11], very few studies conducted to date have explored whether OTA negatively affects embryonic development.

During normal embryogenesis, apoptosis (a unique morphological pattern of cell death) functions to remove abnormal or redundant cells from pre-implantation embryos [12,13]. Apoptotic processes do not occur prior to the blastocyst stage during normal mouse embryonic development [14]. Induction of apoptosis during early stages of embryogenesis (*i.e.*, following exposure to a teratogen) compromises embryonic development [15–19]. Additionally, several chemical and physical triggers of apoptosis create oxidative stress *via* ROS generation [15,20]. This suggests that oxidative stress and apoptosis are closely linked and that ROS generators are potent inducers of apoptosis.

Apoptosis plays an important role in embryonic development [21]. Although several studies have shown that apoptosis is essential for normal embryonic development [22–24], excessive apoptosis triggered in early embryos by exposure to mechanistically diverse teratogens can cause developmental injury [15,16,25–27]. Previous studies found that OTA induced apoptosis in mammalian cells, including monkey and human kidney epithelial cells, porcine kidney PK15 cells, and human OK cells [28–31]. In addition, developmental neurotoxicity caused by OTA was reflected in both morphological and behavioral changes in rodents [32,33]. Pretreatment of cultured post-implantation rat embryos with OTA caused dose-dependent reductions in yolk sac diameter, crown-rump length, somite number count, and protein and DNA content [34]. However, the details of the injuries caused by OTA, and the mechanisms of OTA action, during either the pre- or post-implantation stages of embryonic development, remain unclear. In the present study, we investigated whether OTA exerted cytotoxic effects on early-stage development of mouse blastocysts. We found that OTA suppressed embryonic cell proliferation during the blastocyst stage predominantly by inducing apoptosis of the inner cell mass (ICM). We also monitored subsequent blastocyst development *in vitro* and after embryo transfer *in vivo*.

## 2. Results

### 2.1. Effects of OTA on Mouse Blastocysts

**Figure 1:**
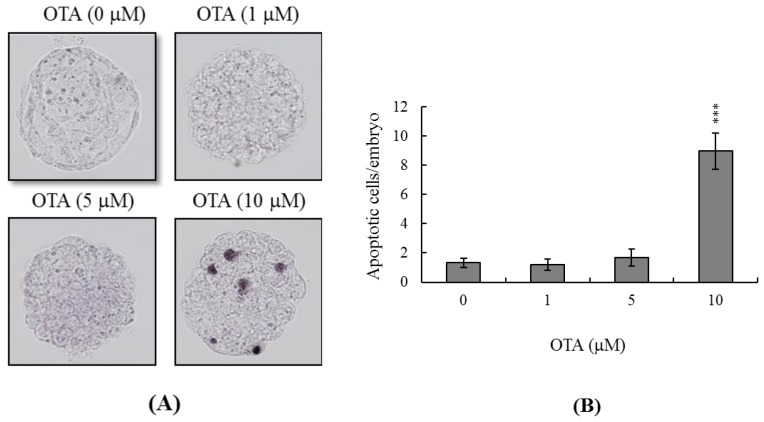
Ochratoxin A (OTA) induces apoptosis in mouse blastocysts. (**A**) Mouse blastocysts were treated with OTA (1, 5, or 10 μM) for 24 h, or left untreated, and the extent of apoptosis was determined using transferase-mediated dUTP nick-end labeling (TUNEL) staining followed by light microscopy. TUNEL-positive cells are shown in black; (**B**) The mean numbers of apoptotic (TUNEL-positive) cells per blastocyst was calculated as six to eight experiments. Data are based on at least 220 blastocysts from each group. Values are presented as means ± SEMs. ***p < 0.001 *versus* the control group.

To explore whether OTA was cytotoxic, we treated mouse blastocysts with 1, 5, or 10 μM OTA at 37 °C for 24 h, and measured apoptosis using the TUNEL assay. OTA at 10 μM clearly induced apoptosis (Figure 1A); the level of cell death was nine-fold higher in OTA-treated blastocysts than in untreated controls (Figure 1B).

### 2.2. Effects of OTA on Cell Proliferation

**Figure 2:**
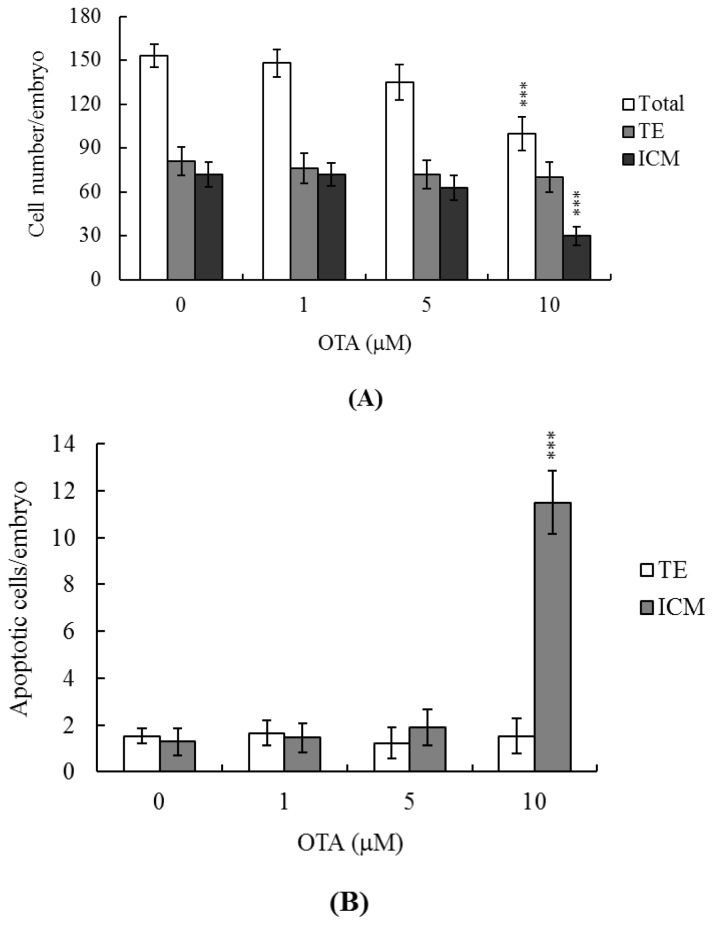
Effects of OTA on blastocyst viability. Mouse blastocysts were treated with OTA (1, 5, or 10 μM) for 24 h, or left untreated. (**A**) The total number of cells per blastocyst, and the numbers of cells in the inner cell mass (ICM) and the trophectoderm (TE) were counted; (**B**) The proportions of Annexin V-positive/PI-negative cells in the blastocysts of each group were determined. Data are based on at least 230 blastocysts from each group. Values are presented as means ± SEMs of five separate experiments. ***p < 0.001 *versus* the control group.

We used differential staining to examine cell proliferation in blastocysts treated with 1, 5, or 10 μM OTA for 24 h, and in untreated controls. The total and ICM cell numbers in blastocysts treated with 10 μM OTA were significantly lower than in controls (Figure 2A). Annexin V and PI staining were used to identify the cell death modes. Significantly higher numbers of Annexin V-positive/PI-negative (apoptotic) cells were evident in the ICM of treated blastocysts compared to controls, but no such difference was apparent in the trophectoderm (TE) (Figure 2B). Thus, OTA induces significant apoptosis in the ICM but not the TE of mouse blastocysts, impairing developmental potential.

### 2.3. Effects of OTA on Mouse Embryonic Developmental Potential *in Vitro*

**Figure 3:**
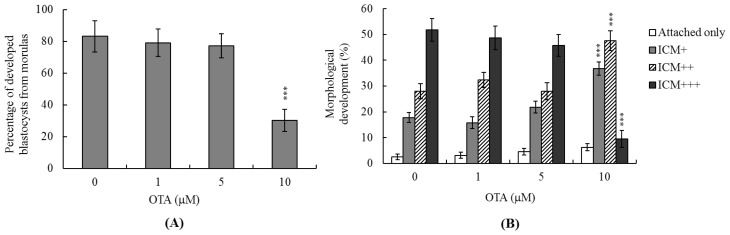
*In vitro* development of mouse embryos exposed to OTA at the blastocyst stage. (**A**) Mouse morulae were treated with OTA (1, 5, or 10 μM) for 24 h, or left untreated, and next cultured for an additional 24 h at 37 °C. The proportions of morulae that developed into blastocysts were counted; (**B**) Mouse blastocysts were treated with OTA (1, 5, or 10 μM) for 24 h, or left untreated, and observed in culture for seven days post-treatment. Growing embryos were classified as either “attached” or “outgrowth,” with the latter defined by the presence of a cluster of inner cell mass (ICM) cells over the trophoblastic layer. ICM clusters were scored according to shape, ranging from compact and rounded ICM (+++) to a few scattered cells (+) over the trophoblastic layer. Blastocysts were identified as exhibiting attachment only, or as ICM (+), ICM (++), or ICM (+++), via morphological assessment, as described in Materials and Methods. Values are presented as means ± SEMs of six experiments. ***p < 0.001 *versus* the control group.

We next analyzed the effects of OTA on embryonic pre-implantation, implantation, and post-implantation development *in vitro*. Untreated control morulae developed into blastocysts at a frequency of ~83%, compared to only 30.4% of morulae treated with 10 μM OTA (Figure 3A). To explore the effects of OTA on implantation and post-implantation events *in vitro*, blastocysts were treated with 1, 5, or 10 μM OTA (300–320 blastocysts in each group), or remained untreated (control blastocysts), and the implantation rate and subsequent development over 8 days in culture were analyzed. The implantation rate of and the extent of attachment to fibronectin-coated dishes were similar in the OTA-treated and control groups (Figure 3B). Importantly, OTA-pretreated blastocysts were less successful in achieving post-implantation developmental milestones than were control blastocysts (Figure 3B), indicating that OTA affects the *in vitro* potential of blastocysts to develop features characteristic of post-implantation embryos.

### 2.4. Effects of OTA on the Developmental Potential of Blastocysts *in Vivo*

**Figure 4:**
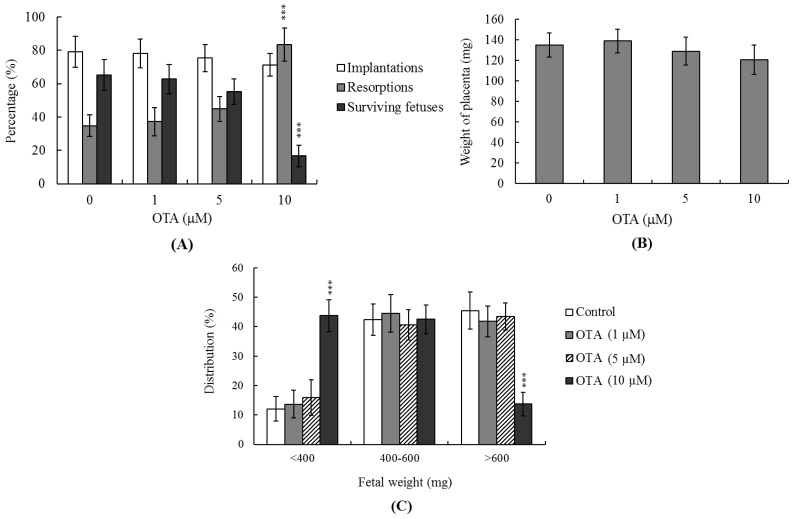
Effects of OTA on mouse blastocysts in terms of *in vivo* implantation, resorption, fetal survival and fetal weight. (**A**) Mouse blastocysts were treated with OTA (1, 5, or 10 μM) for 24 h, or left untreated. Implantation, resorption, and numbers of surviving fetuses, were analyzed, as described in Materials and Methods. The implantation proportions represent the number of implantations per transferred embryo, ×100. The proportions of resorption or fetal survival are the numbers of resorptions or surviving fetuses per-implantation, ×100; (**B**) Placental weights were measured in each of 40 recipient mice; (**C**) Weight distribution of surviving fetuses on day 18 post-coitus. Surviving fetuses were obtained *via* embryo transfer of control and OTA-pretreated blastocysts, as described in Materials and Methods (a total of 320 blastocysts were transferred to 40 recipients). ***p < 0.001 *versus* the OTA-free group. 2.5.

To further explore the effects of OTA on blastocyst development *in vivo*, we performed embryo transfers on mouse blastocysts pretreated with OTA or not, and examined uterine contents 13 days post-transfer (day 18 fetus). The implantation ratio of the group pretreated with OTA (10 μM) was not significantly different from that of the untreated control group (Figure 4A). Embryos that implanted but failed to develop were subsequently resorbed. However, the proportion of such embryos was significantly higher in the group pretreated with 10 μM OTA compared to the control group (Figure 4A). Interestingly, no difference in placental weight was evident between the OTA-treated and untreated groups (Figure 4B), but fetal weight was lower in the OTA-treated group. Moreover, both earlier and current experiments by our group have shown that 35%–40% of normal mouse fetuses weigh more than 600 mg at day 18 of pregnancy after mouse embryo transfer, and the average weight of all surviving fetuses was ~600 ± 12 mg in the untreated control group of the present experiment [16,17,35]. Fetal weight is an important indicator of developmental status, and the average fetal weight of untreated controls thus serves as a key indicator of the developmental status of OTA-treated blastocysts. Only about 14% of fetuses in the group pretreated with 10 μM OTA weighed over 600 mg, in contrast to 45.5% of control fetuses (Figure 4C). Thus, exposure to OTA at the blastocyst stage is associated with a risk of poor post-implantation development.

### 2.5. ROS Generation and Mitochondrion-Dependent Apoptotic Processes Are Involved in Blastocyst Death Induced by OTA

**Figure 5:**
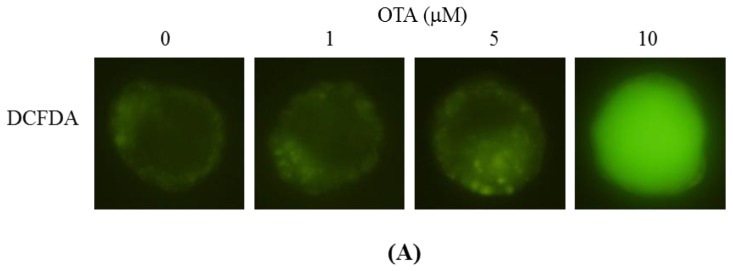

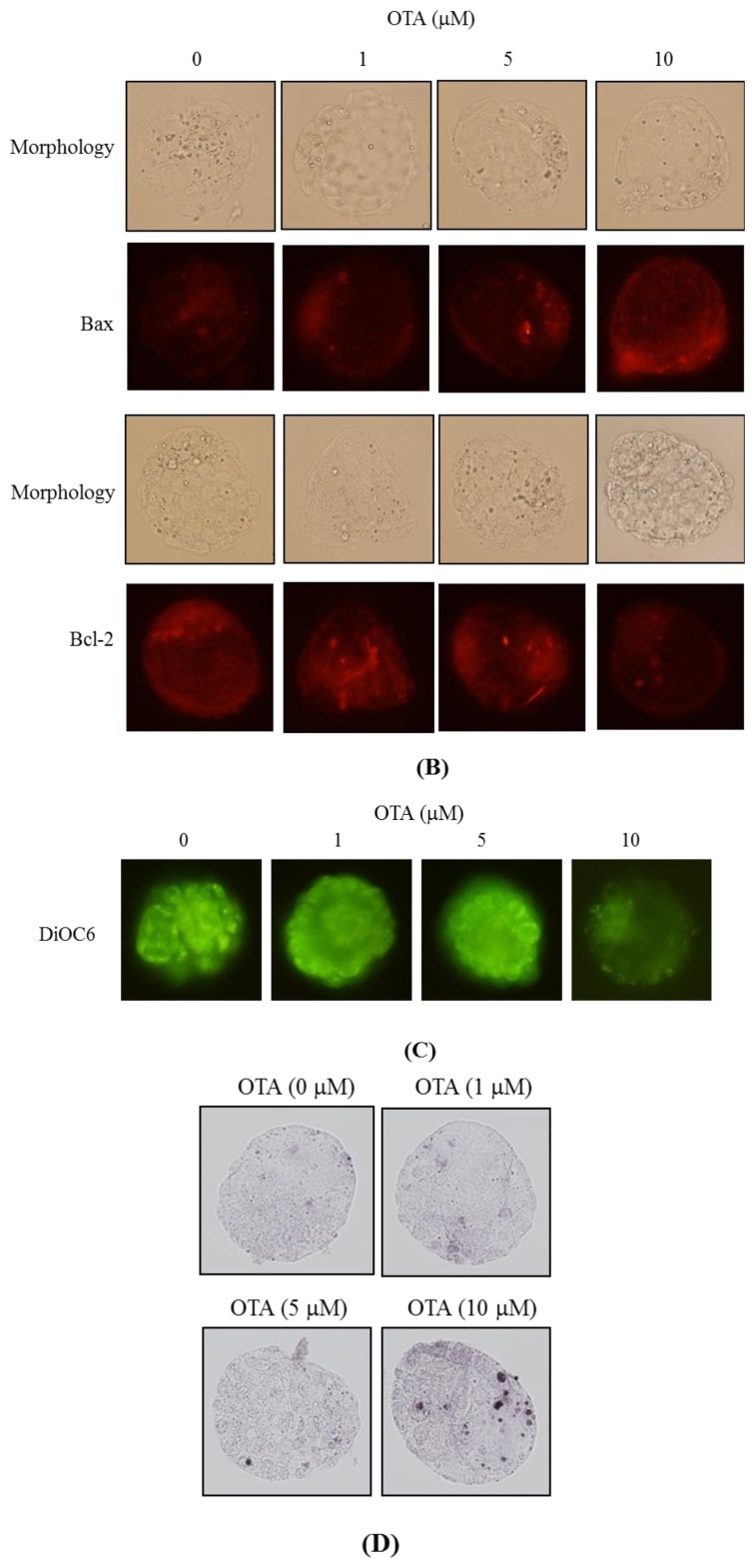
Effects of OTA on ROS generation and mitochondrion-dependent apoptotic processes in mouse blastocysts. Mouse blastocysts were treated with OTA (1, 5, or 10 μM), or left untreated, for 24 h. (**A**) ROS generation was detected by staining with a 20 μM solution of the DCF-DA fluorescent dye; (**B**) Bax and Bcl-2 expression levels were assessed *via* immunostaining using anti-Bax and anti-Bcl-2 antibodies, respectively. The protocol is described in Materials and Methods; (**C**) To determine changes in mitochondrial membrane potential, embryos were incubated with 40 nM DiOC6(3) at 37 °C for 1 h and examined under a fluorescence microscope; (**D**) Activation of caspase-3 was measured by immunostaining with anti-activated caspase-3 antibody for 3 h, followed by addition of a secondary antibody conjugated with peroxidase (1:100 dilution) for 1 h. Finally, 20 μL of DAB-substrate solution was added to each embryo, and incubation for 2 min at room temperature followed. Cells containing activated caspase-3 are shown in black.

**Figure 6:**
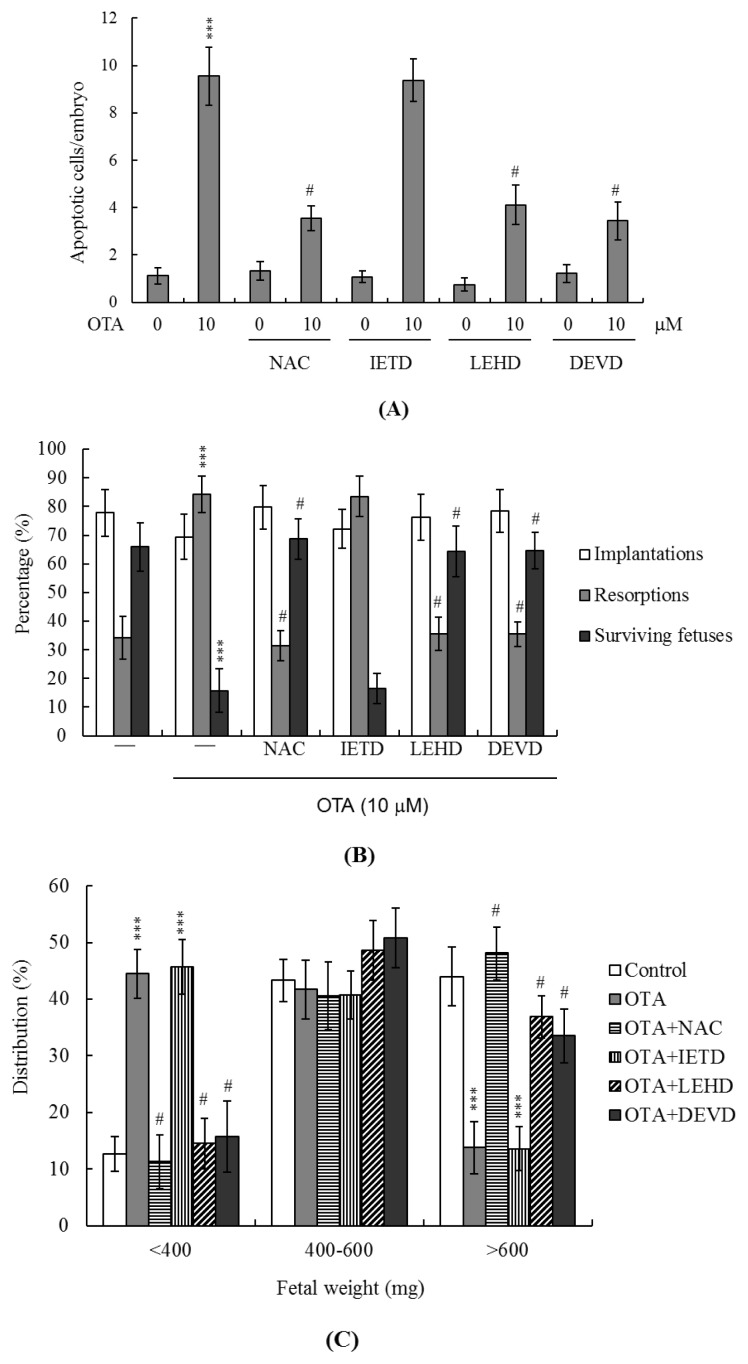
Effects of ROS scavengers and caspase inhibitors on *in vivo* implantation, resorption, fetal survival, and fetal weight, after treatment of embryos with OTA. Mouse blastocysts were pretreated with 400 μM *N*-acetyl cysteine (NAC), 300 μM Z-IETD-FMK (IETD), 300 μM Z-LEHD-FMK (LEHD), or 300 μM Z-DEVD-FMK (DEVD) for 1 h, or left untreated. Blastocysts were incubated with 10 μM OTA for a further 24 h. (**A**) Apoptosis was detected *via* TUNEL staining, as described in the legend to Figure 1; (**B**) The extent of implantation and resorption, and the numbers of surviving fetuses, were analyzed *via* embryo transfer, as described in Materials and Methods and in the legend to Figure 4; (**C**) The weight distribution of surviving fetuses on day 18 post-transfer. Surviving fetuses were obtained *via* embryo transfer of control and OTA-pretreated blastocysts (a total of 320 blastocysts were transferred to 40 recipients). ***p < 0.001 *versus* the OTA-free group and # *p* < 0.001 *versus* the group treated with 10 μM OTA.

In light of the data of previous reports and our recent finding that ROS effectively induce apoptosis [16,36,37], we used the fluorescent dye DCF-DA to measure ROS content in OTA-treated mouse blastocyst cells. As shown in Figure 5A, OTA at 10 μM directly induced an increase in fluorescence intensity in such cells, compared with that of untreated control cells. Changes in the expression levels of Bax and Bcl-2 affect the action of the mitochondrion-dependent apoptotic pathway [38,39]; high and low Bax/Bcl-2 ratios are associated with lower and higher apoptotic thresholds, respectively. We thus explored whether OTA induced apoptosis *via* modulation of Bax and Bcl-2 expression. Immunostaining revealed that the Bax and Bcl-2 levels increased and decreased, respectively, upon OTA treatment of mouse blastocysts (Figure 5B). Examination of the effect of OTA on the mitochondrial membrane potential (MMP) of mouse blastocyst cells revealed that treatment with 10 μM OTA suppressed DiOC_6_(3) uptake into mitochondria, indicative of significant loss of MMP (Figure 5C). In addition, 10 μM OTA significantly activated caspase-3; this is an important feature of apoptosis (Figure 5D). To further explore the roles played by ROS and apoptosis-associated events in OTA-induced apoptosis, we added a recognized ROS scavenger, *N*-acetyl cysteine (NAC), and various caspase-specific inhibitors, to OTA-treated mouse blastocysts. Pretreatment of cells with NAC (500 μM) attenuated OTA-induced apoptosis (Figure 6A). In addition, pretreatment with inhibitors specific for caspase-9 (Z-LEHD-FMK) and caspase-3 (Z-DEVD-FMK) effectively blocked apoptosis, whereas the caspase-8-specific inhibitor Z-IETD-FMK did not (Figure 6A). Importantly, treatment with 10 μM OTA was associated with a lower implantation ratio, and the failure of further development was effectively blocked by addition of NAC and inhibitors of caspase-9 and caspase-3 at the time of embryo transfer. Animals pretreated with the caspase-8 inhibitor were similar in all respect to untreated controls (Figure 6B). In addition, the lower fetal weight in the group treated with 10 μM OTA was effectively rescued by pretreatment with NAC and specific caspase-9 and -3 inhibitors (Figure 6C). We thus suggest that OTA triggers ROS generation, in turn activating mitochondrion-dependent apoptotic processes in mouse blastocyst cells.

## 3. Discussion

Chemical or physical injury can affect normal progression of the complex and precisely orchestrated process of embryonic development, leading to malformation or miscarriage of the embryo. It is thus important to explore the possible teratogenic effects of various chemical agents and environmental toxins. Several previous reports have shown that OTA is a neurotoxic mycotoxin that causes oxidative stress, DNA damage, and mitochondrial dysfunction [10,40]. OTA-induced oxidative DNA damage has been found in the brain [6], and OTA increased oxidative stress in neural stem/progenitor cells [41]. A recent study found that OTA compromised hippocampal neurogenesis *in vivo* and that such injury might be associated with memory loss and deficits in learning and memory [41]. OTA not only induces neurotoxicity, but also kills other types of cells. Thus, exposure to OTA triggers caspase-dependent apoptosis *via* the mitochondrial pathway in human hepatocarcinoma cells [42] and disturbs calcium flux, causing DNA damage and apoptosis, in porcine kidney PK15 cells [30]. Thus, OTA may be a teratogen if ingested during pregnancy. Several papers have shown the teratogenic effects of OTA in mouse, rat, and pig [43–46]. However, the regulatory effects and related mechanisms of OTA-induced cytotoxicity on early embryonic development have not been studied in detail. We therefore employed an *in vitro* assay to assess the cytotoxic effects of OTA during embryonic development, and sought to explain the mechanism of action of the chemical. Preliminary experiments revealed that OTA triggered apoptosis of mouse blastocysts only when the cells were incubated with OTA for at least 12 h; the effects endured for 24 h (data not shown). We therefore incubated blastocysts in medium containing 1–10 μM OTA for 24 h. Cell numbers fell as apoptosis developed (Figure 1). TUNEL staining revealed that treatment of mouse blastocysts with 10 μM OTA induced an 8.9-fold rise in apoptosis (Figure 1). Further, dual differential and Annexin V staining showed that the OTA-induced apoptosis occurred primarily in the ICM (Figure 2).

The TE arises from the trophoblast at the blastocyst stage and develops into a sphere of epithelial cells surrounding the ICM and the blastocoel. These cells contribute to placenta formation and are required for development of the mammalian conceptus [47]. Thus, reduction in the numbers of cells in the TE lineage may reduce both implantation and embryonic viability [48,49]. However, in our experiments, OTA induced apoptosis only in the ICM, thus not in the TE, and did not deleteriously affect embryonic attachment or outgrowth either *in vitro* or *in vivo* (Figures 2, 3 and 4). Previous studies have shown that a reduction of ~30% or more in the number of ICM cells is associated with a high risk of fetal loss or developmental injury, even when the implantation rate and TE cell numbers are normal [50]. In addition, high numbers of ICM cells are essential to ensure viable implantation, and a reduction in cell numbers may decrease embryonic viability [48,49,51]. However, we found, in the present work, that OTA-induced embryonic cell death occurred only in the ICM, compromising post-implantation development, but exerting no effect on implantation per se (Figures 2, 3 and 4). Although apoptosis is employed to eliminate unwanted cells during normal embryonic development, this process does not normally occur at the blastocyst stage [12,13]. Apoptotic action before or during blastocyst development is likely to result in deletion of important cell lineages, thus affecting embryonic development and potentially leading to miscarriage or embryonic malformation [14]. Our coworkers are currently using primary cell cultures derived from mouse ICM and TE to examine the negative effects of OTA on these cells, and to begin investigating the underlying mechanisms. Their preliminary results have revealed that OTA induces ICM cell apoptosis at concentrations higher than 10 μM. Low doses of OTA (<5 μM) had no injury effects in TE cell lines, whereas high concentrations (20–40 μM) triggered apoptosis. Notably, OTA appears to induce apoptosis in both mouse ICM and TE cell cultures through the mitochondria-dependent apoptotic pathway, as evident from increases in the Bax/Bcl-2 ratio and decreases in mitochondrial membrane potential. Accordingly, we conclude that OTA induces apoptosis in the ICM and TE *via* the same regulatory mechanism, but the treatment dose required to induce negative effects can vary by cell type. In view of our findings that OTA reduced cell number and increased apoptosis specifically in the ICM of mouse blastocysts, we determined whether OTA affected embryonic implantation and mortality, and/or caused developmental delay in post-implantation mouse embryos either *in vitro* or *in vivo* (Figure 2). We found that OTA-treated blastocysts exhibited a reduced level of embryonic development and a higher level of embryonic death both *in vitro* and *in vivo* (Figures 3 and 4).

Moreover, OTA has been shown to induce cell death with an IC_50_ value of 14 μM in porcine kidney PK15 cells [30]. Preliminary experiments by our group further revealed that OTA dose-dependently triggers mouse embryonic stem cell (ESC) apoptosis with an IC_50_ value of 12.7 μM, as determined by MTT assays performed after 24 h of exposure (data not shown). In addition, our initial HPLC results showed that serum OTA levels were about 9.6 μM in mice that had been exposed to drinking water supplemented with 20 μM OTA over 4 days (data not shown). Our embryo transfer assays showed that the 13-days-post-transfer (day 18 fetus) fetal weight of the OTA-pretreated group (10 μM) was significantly lower than that of the untreated control group (Figure 4C). However, we did not perform immunohistochemical staining of developmental markers in various organs of day 18 fetuses. To further determine the impact of OTA on early embryonic development in a stem cell assay model, our coworkers recently incubated cells with or without OTA and examined their ability to form embryoid bodies *in vitro*. Embryoid body formation was significantly decreased in cells pretreated with OTA. To ascertain whether the expression levels of OCT 4 and phosphorylated STAT3 (two well-known pluripotent markers) are affected by OTA, stem cells were treated with or without OTA for 24 h. Immunoblotting experiments revealed that OTA had no significant effect on the expression levels of OCT 4 or phosphorylated STAT3, compared to the untreated control group. Moreover, treatment of embryoid body cells with 50 ng/mL nerve growth factor (NGF) for 14 days induced their differentiation into nerve cells, as reflected by expression of microtubule associated protein-2 (MAP-2), a major nerve cell biomarker. Notably, pre-treatment with 10 μM OTA effectively inhibited the NGF-induced expression of MAP-2 (data not shown). Collectively, these results suggest that OTA inhibits early embryonic development and may have injury effects on neuronal organs. These experimental data showed that OTA induces apoptosis and negatively affects mouse embryonic development both *in vitro* and *in vivo*. Thus, OTA appears to compromise both pre- and post-implantation embryonic development at doses that are physiological, in the sense that they may be attained *via* dietary intake.

Mechanistically, our data show that OTA directly evokes intracellular oxidative stress (Figure 5A) leading to ROS-mediated apoptosis of mouse blastocyst cells (Figure 6A). This seems to involve the mitochondrion-dependent apoptotic pathway, as indicated both by the OTA-induced changes in the intracellular levels of Bcl family members (Bax and Bcl-2) and the loss of mitochondrial membrane potential (Figure 5B). Our findings are consistent with those of previous studies showing that OTA could trigger the mitochondrion-dependent apoptotic process, as revealed by loss of mitochondrial transmembrane potential, increased ROS production, mitochondrial relocalization of Bax, release of cytochrome c, and activation of caspases [10]. Given that recent studies have shown that addition of specific compounds to commonly used cell culture media triggers generation of ROS, such as hydrogen peroxide [52,53], we co-incubated OTA and culture medium, and measured ROS levels using the ferrous iron oxidation-xylenol orange method [52]. No artifactual ROS generation was detected under such conditions (data not shown). Importantly, a well-known ROS scavenger, NAC, effectively prevented OTA-induced apoptosis in mouse blastocysts (Figure 6A). We explored the precise mechanism of OTA-induced apoptosis in such blastocysts (Figures 5 and 6). NAC is not suitable for use in our animal model due to the high dose (400 μM) that is required to prevent OTA-induced embryonic development injury *in vitro* (Figure 6). However, we observed that pretreatment with NAC (400 μM) did not fully prevent OTA-induced cell apoptosis in mouse blastocysts (Figure 6A). This suggests that OTA may have a dual mode of action to cause cell apoptosis, such as through ROS generation and the formation of DNA adducts, potentially explaining why the antioxidant only partially inhibited OTA-induced apoptosis in mouse blastocyst-stage embryos. Although additional studies will be required to assess this possibility in detail, our present work provides important new insights into the negative impact of OTA on early embryonic development.

A recent study showed that OTA treatment of human peripheral blood mononuclear cells triggered the release of reactive oxygen species (ROS) and increased the levels of 8-hydroxydeoxyguanosine (8-OHdG), an important biomarker of oxidative DNA stress [54]. Investigation further showed that OTA treatment of human peripheral blood mononuclear cells *in vitro* could induce cell cycle arrest at G1 phase by down-regulating the protein expression levels of CDK4 and cyclinD1, and also trigger cell apoptosis. These results demonstrated that ROS is involved in OTA-induced DNA damage and G1 arrest in human peripheral blood mononuclear cells, suggesting that OTA may negatively affect embryonic development in mouse blastocysts *via* ROS-induced DNA damage. Our results collectively show that OTA triggers apoptosis of the ICM cells of blastocysts, leading to impairment of embryonic development *via* ROS generation, which in turn stimulates various adverse downstream processes characteristic of the mitochondrion-dependent apoptotic pathway.

A previous study further found that OTA has a high potential to initiate skin tumors in mouse skin *in vivo*, acting through oxidative stress, MAPK signaling and DNA damage. The same study also found that anti-oxidants may help prevent OTA-induced tumorigenesis [55]. Moreover, OTA has been shown to inhibit cell proliferation and downregulate heat shock protein 70 and 27 in cultured human hepatocellular carcinoma cells, but without inducing significant generation of reactive oxygen species [42]. DNA damage following OTA treatment of cultured human hepatocellular carcinoma cells induced apoptosis *via* the p53 protein, which triggers mitochondria- and caspase-dependent apoptotic processes. The p53 protein directly interacts with BCL-2 family proteins; this permits mitochondrial outer membrane permeabilization, leading to the release of apoptogenic proteins, such as cytochrome c, which activate caspases to trigger the cell death cascade. In addition, none oxidant, OTA seems to be genotoxic, triggering mitochondrial- and caspase-dependent apoptosis. From these results, we conclude that OTA may inhibit the transcriptional process. However, oxidative damage is not a major contributor to OTA toxicity in human hepatocellular carcinoma cells [42]. Based on the prior results and our present data, we speculate that OTA may have dual means to cause apoptosis, such as through ROS generation and the formation of DNA adducts. This would explain why antioxidants only partially inhibit OTA-induced apoptosis in mouse blastocyst-stage embryos (Figure 6A). Thus, although future work will be required to examine the detailed mechanisms of this process, our present study provides important new insights into the negative impacts of OTA on early embryonic development.

## 4. Experimental Section

### 4.1. Materials

Ochratoxin A (OTA), Pregnant mare’s serum gonadotropin (PMSG), Bovine serum albumin (BSA), sodium pyruvate and puerarin were purchased from Sigma (St. Louis, MO, USA). Human chorionic gonadotropin (hCG) was obtained from Serono (NV Organon Oss, The Netherlands). The TUNEL in situ cell death detection kit was obtained from Roche (Mannheim, Germany) and CMRL-1066 medium was from Gibco Life Technologies (Grand Island, NY, USA). Z-DEVD-FMK, Z-LEHD-FMK and Z-IETD-FMK were from Calbiochem (La Jolla, CA, USA).

### 4.2. Collection of Mouse Morulas and Blastocysts

ICR mice were from National Laboratory Animal Center (Taiwan, ROC). This research was also approved by the Animal Research Ethics Board of Chung Yuan Christian University (Taiwan, ROC). All animals received humane care, as outlined in the Guidelines for Care and Use of Experimental Animals (Canadian Council on Animal Care, Ottawa, 1984). All mice were maintained on breeder chow (Harlan Teklad chow) with food and water available *ad libitum*. Housing was in standard 28 cm × 16 cm × 11 cm (height) polypropylene cages with wire-grid tops and kept under a 12 h day/12 h night regimen. Nulliparous females (6–8 weeks old) were superovulated by injection of 5 IU PMSG followed 48 h later by injection of 5 IU hCG, and then mated overnight with a single fertile male of the same strain. The day a vaginal plug was found was defined as day 0 of gestation. Plug-positive females were separated for experimentation. Morulas were obtained by flushing the uterine tubes on the afternoon of gestation day 3, and blastocysts were obtained by flushing the uterine horn on day 4; in both cases the flushing solution consisted of CMRL-1066 culture medium containing 1 mM glutamine and 1 mM sodium pyruvate. Expanded blastocysts from different females were pooled and randomly selected for experiments.

### 4.3. OTA Treatment and TUNEL Assay

Blastocysts were incubated in medium containing with or without 1, 5, or 10 μM OTA for 24 h. For apoptosis detection, embryos were washed in OTA-free medium, fixed, permeabilized and subjected to TUNEL labeling using an *in situ* cell death detection kit (Roche Molecular Biochemicals, Mannheim, Germany) according to the manufacturer’s protocol. Photographic images were taken under brightfield illumination using a fluorescence microscope (Olympus BX70, Tokyo, Japan).

### 4.4. OTA Treatment and Cell Proliferation

Blastocysts were incubated with or without culture medium containing indicated concentrations of OTA (1–10 μM) for 24 h. Then, blastocysts were washed with OTA-free medium and dual differential staining was used to facilitate counting of cell numbers in the inner cell mass (ICM) and trophectoderm (TE) [48]. Blastocysts were incubated in 0.4% pronase in M_2_-BSA medium (M_2_ medium containing 0.1% bovine serum albumin) for removal of the zona pellucida. The denuded blastocysts were exposed to 1 mM trinitrobenzenesulphonic acid (TNBS) in BSA-free M_2_ medium containing 0.1% polyvinylpyrrolidone (PVP) at 4 °C for 30 min, and then washed with M_2_ medium (Sigma, St. Louis, MO, USA) [56]. The blastocysts were further treated with 30 μg/mL anti-dinitrophenol-BSA complex antibody in M_2_-BSA at 37 °C for 30 min, and then with M_2_ medium supplemented with 10% whole guinea-pig serum as a source of complement, along with 20 μg/mL bisbenzimide and 10 μg/Ml propidium iodide (PI), at 37 °C for 30 min. The immunolysed blastocysts were gently transferred to slides and protected from light before observation. Under UV light excitation, the ICM cells (which take up bisbenzimidine but exclude PI) appeared blue, whereas the TE cells (which take up both fluorochromes) appeared orange-red. Since multinucleated cells are not common in pre-implantation embryos [57], the number of nuclei was considered to represent an accurate measure of the cell number. Photographic images were taken under a fluorescence microscope (Olympus IX71, Tokyo, Japan).

### 4.5. Annexin V Staining

Blastocysts were incubated in 0, 1, 5 or 10 μM OTA for 24 h, washed with OTA-free culture medium, and then stained using an Annexin V-FLUOS staining kit (Roche, Mannheim, Germany), according to the manufacturer’s instructions. Briefly, the blastocysts were incubated in M_2_-BSA for removal of the zona pellucida, washed with PBS plus 0.3% BSA, and then incubated for 60 min with a mixture of 100 μL binding buffer, 1 μL fluorescein isothiocyanate (FITC)-conjugated Annexin V and 1 μL PI. After incubation, the embryos were washed and photographed using a fluorescence microscope under fluorescent illumination. Cells staining Annexin V+/PI− were considered apoptotic, while those staining Annexin V+/PI+ were considered necrotic.

### 4.6. Morphological Analysis of Embryonic Development

Blastocysts were cultured according to a modification of the previously reported method [58]. Briefly, embryos were cultured in 4-well multidishes at 37 °C. For group culture, four embryos were cultured per well. The basic medium consisted of CMRL-1066 supplemented with 1 mM glutamine and 1 mM sodium pyruvate plus 50 IU/mL penicillin and 50 mg/mL streptomycin (hereafter called culture medium). For treatments, the embryos were incubated with 0, 1, 5 or 10 μM OTA for 24 h. Thereafter, the embryos were cultured for 3 days in culture medium supplemented with 20% fetal calf serum, and for 4 days in culture medium supplemented with 20% heated-inactivated human placental cord serum, for a total culture time of 8 days from the onset of treatment. Embryos were inspected daily under a phase-contrast dissecting microscope, and developmental stages were classified according to established methods [59,60]. Under these culture conditions, each hatched blastocyst attached to the fibronectin and grew to form a cluster of ICM cells over the trophoblastic layer *via* in a process called TE outgrowth. After a total incubation period of 96 h, morphological scores for outgrowth were estimated. Growing embryos were classified as either “attached” or “outgrowth”, with the latter defined by the presence of a cluster of ICM cells over the trophoblastic layer. As described previously [61,62], ICM clusters were scored according to shape, ranging from compact and rounded ICM (+++) to a few scattered cells (+) over the trophoblastic layer.

### 4.7. Blastocyst Development following Embryo Transfer

To examine the ability of expanded blastocysts to implant and develop *in vivo*, the generated embryos were transferred to recipient mice. ICR females (white skin color) were mated with vasectomized males (C57BL/6J; black skin color; from National Laboratory Animal Center, Taiwan, ROC) to produce pseudopregnant dams as recipients for embryo transfer. To ensure that all fetuses in the pseudopregnant mice came from embryo transfer (white color) and not from fertilization by C57BL/6J (black color), we examined the skin color of the fetuses at day 18 post-coitus. To assess the impact of OTA on post-implantation growth *in vivo*, blastocysts were exposed to 0, 1, 5 and 10 μM OTA for 24 h, and then 8 embryos were transferred in parallel to the paired uterine horns of day 4 pseudopregnant mice. The surrogate mice were killed on day 18 post-coitus, and the frequency of implantation was calculated as the number of implantation sites per number of embryos transferred. The incidence rates of resorbed and surviving fetuses were calculated as the number of resorptions or surviving fetuses, respectively, per number of implantations. The weights of the surviving fetuses and placentae were measured immediately after dissection.

### 4.8. Immunofluorescent Cell Stain

Mouse blastocyst cells were fixed by formaldehyde, permeabilized by 1% Triton X-100, blocked by bovine serum albumin (5 mg/mL in PBS), and incubated with anti-Bax or anti-Bcl-2 antibodies (40 mg/mL) at room temperature for 3 h. After washing three times with PBS, embryos were incubated with second antibody conjugated with FITC or Rhodamine (TRITC) (1:100) at room temperature for 1 h and then observed under a fluorescence microscope (Olympus BX70, Tokyo, Japan).

### 4.9. Statistics

The data were analyzed using one-way ANOVA and *t*-tests and are presented as the mean ± SEM, with significance at *p* < 0.05.

## 5. Conclusions

In summary, for the first time, we have shown that OTA induces apoptosis in the ICM (only) of mouse blastocysts via ROS- and mitochondrion-dependent pathways, decreasing embryonic development and viability. OTA is thus potentially hazardous to normal embryonic development. Further studies are required to determine the effects of dietary OTA on embryonic development during pregnancy, and to define the precise regulatory mechanism(s) whereby OTA affects embryonic development, possibly acting as a teratogen during human embryogenesis.
